# Animal Hairs as Water-stimulated Shape Memory Materials: Mechanism and Structural Networks in Molecular Assemblies

**DOI:** 10.1038/srep26393

**Published:** 2016-05-27

**Authors:** Xueliang Xiao, Jinlian Hu

**Affiliations:** 1 Key Laboratory of Eco-Textiles, Ministry of Education, Jiangnan University, Wuxi, P.R. China; 2 Institute of Textiles and Clothing, the Hong Kong Polytechnic University, Hong Kong, China

## Abstract

Animal hairs consisting of α-keratin biopolymers existing broadly in nature may be responsive to water for recovery to the innate shape from their fixed deformation, thus possess smart behavior, namely shape memory effect (SME). In this article, three typical animal hair fibers were first time investigated for their water-stimulated SME, and therefrom to identify the corresponding net-points and switches in their molecular and morphological structures. Experimentally, the SME manifested a good stability of high shape fixation ratio and reasonable recovery rate after many cycles of deformation programming under water stimulation. The effects of hydration on hair lateral size, recovery kinetics, dynamic mechanical behaviors and structural components (crystal, disulfide and hydrogen bonds) were then systematically studied. SME mechanisms were explored based on the variations of structural components in molecular assemblies of such smart fibers. A hybrid structural network model with single-switch and twin-net-points was thereafter proposed to interpret the water-stimulated shape memory mechanism of animal hairs. This original work is expected to provide inspiration for exploring other natural materials to reveal their smart functions and natural laws in animals including human as well as making more remarkable synthetic smart materials.

Shape-memory polymers (SMP) have fascinated scientists for decades. Most advances of SMP outputs can be found in many review papers^1^,^2^,^3^,^4^,^5^. Take water-stimulated SMP as an example, this kind of polymer shows an ability of shape recovery from a temporary shape to the original/predetermined shape when water stimulus is applied after SMP being quasi-plastically distorted by means of altering their internal physical properties^1^. To date, a number of water-triggered synthetic hybrid SMPs have been reported^6^,^7^,^8^,^9^, such as shape memory polyurethanes filled with cotton cellulose nanowhiskers, cupric sulphate pentahydrate, pyridine moieties, poly(vinyl alcohol) and chitosan with polyethylene glycol, respectively. Even more, co-block synthetic polymers were also reported to have water-stimulated shape memory effect (SME), such as the copolymerization of polyhedral oligomeric silsesquioxane molecules and poly (ethylene glycol) for hard and soft segments separately, or N-bis(2-hydroxylethyl) isonicotinamine and hexamethylene diisocyanate plus with 1.4-butanediol with certain content ratios for moisture sensitive SME^10^,^11^,^12^, etc. Their water-active SME was disclosed due to the plasticizing effect of water molecules on polymeric materials, thus to increase the flexibility of macromolecule chains of SMPs^13^,^14^. Hydrophilic group or water-soluble ingredient sounds compulsory in the water-stimulated SMP compounds. A common structure feature of water-stimulated SMP was concluded with net-points and switch unit, as shown in Fig. 1a.

The net-points determine the original/permanent shape of SMPs, and are made of hydrophobic group in the format of either chemical cross-links, crystals, or interlocked supramolecular complex^1^. These groups connect macromolecule chains in the form of network that cannot be interrupted by aqueous polar molecules. The driving force for shape recovery of SMPs is the entropic elasticity of the polymeric network locked by the net-points. The switch unit off and on under removal and penetration of aqueous molecules from/into SMPs is responsible for controlling the shape temporary fixity and shape recovery along with the wetting and drying processes^15^, as shown a typical SME program in Fig. 1b. The aforementioned synthetic SMPs have both structural components by introducing switch unit (hydrophilic group into co-block chains) into the existing polymeric chains, or assembling net-points and switches using copolymerization of relative monomers.

In a SMP system, the elastic recovery force from physical/chemical net-points and intermolecular forces from attractive and/or covalent bonds as switch are in necessity. Fig. 1 demonstrates the design rule and work principle for the synthetic SMP systems. In the last five years, natural fibers and bio-inspired polymers based on the β-folded protein chains of spider silk as net-point^16^,^17^, or coating SMP finishing agent on the natural fibers^18^ for SME were reported. In analogous manner to synthetic/semi-natural shape memory materials, many natural biological materials, in particular biopolymers, are capable of undergoing intelligent adaptations and responses to environmental stimuli^19^,^20^,^21^,^22^ through long-period evolution/selection under ecological conditions^23^,^24^. For instance, peacock tail convert feather containing β-folded protein chains in rachis was discovered recently with evident water sensitive SME^25^, indicating the ability of pure protein fibers for water sensitive SME. In this article, we focus on the investigation of water-stimulated SME of α -helical keratin biopolymer fibers, such as animal hair fibers, which have not been specifically reported yet.

Conventionally, animal hair fibers are merely regarded as ideal raw textile materials because of their excellent comfort, lustre, elasticity and thermal insulation. Higher moisture regain value (~15%) of animal hairs than other natural fibers^26^,^27^ indicates a large amount of hydrophilic groups on and inside the fiber compared with the β-folded protein and cellulose-type fibers. Moreover, the high moisture regain of hair fibers are also ascribed to the hierarchical structure^28^ of cortex with the macro- and micro- fibrils and helical coils, as well as the fiber porous medulla and outside super-thin cuticle scales (1 μm thickness).

Regarding the interaction of aqueous molecules with animal hair, Speakman^29^ was the first to note the neglected longitudinal swelling (1.2%) and evident diametral swelling (16%) of wool over the same change of moisture content from dry to wet, suggesting that the wool fiber was made of a structure more rigid axially than molecular chains swelling laterally. Feughelman^30^,^31^ then proposed a two-phase model for wool, in which the long, water-impenetrable relative rigid cylindrical rods (phase C in Fig. 2) represent the crystalline phase (fibrils), are parallel to the fiber axis and embedded in a water-absorbing matrix (phase M in Fig. 2).

It was justified^32^ the water-impenetrable crystalline component with less than 2% (negligible) in 16% of diametral swelling of wool in water using X-ray diffraction. Fraser and MacRae^33^ confirmed the phase C with ordered crystalline component of microfibrils in the wool cortex from polarized infrared absorption measured data. Astbury^34^,^35^ proposed the transition of organized folded polypeptide chains in α-helical form to be extended β-sheet structure when wool is on high extension in water. Based on this, Pauling^36^ proposed that the α-helical folded structure is formed by the interaction of hydrogen bonds (HBs) between the amide N-H and carboxylic C=O groups of successive turns of each helix, and Fraser *et al.*^37^ proposed that phase M is rich in high-sulfur forming glycine tyrosine and cystine proteins. Under dry state, phase C of wool fiber accounts for 25~30% volume of the whole fiber indicated by mechanical, infrared and X-ray diffraction data^38^. Under wet state, the high-sulfur protein, high-glycine tyrosine proteins, non- helical water-penetrable materials, and the absorbed water in wool are all considered as phase M.

Consistent with the two-phase model, Eaves *et al.*^39^ found that the *T*_*g*_ of wool is decreased with the increase of absorbed water for the cause of disruption of HBs by water molecules in phase M, leading to the increased mobility of macromolecular chains but maintained intact of the chemical cross-linking and crystals (net-work). The residual groups of the collapsed HBs can be re-formed when the hair fiber is dried^40^, which indicates the reversible HBs with and without water alternatively. This switch nature of the biopolymers that people never notice before is a typical condition for SME triggered by water, which renders the smart behavior of hair fibers.

This article, based on the two-phase model of wool, attempts to understand the SME mechanism of animal hair fibers responsive to water. Three kinds of hair fibers from sheep, goat, and camel are investigated for their water-induced SME ability. The relative net-points and switch units are characterized and identified based on their water-stimulated SME respectively.

## Results and Discussion

### Swelling and water-stimulated SME of animal hair fibers

Each hair fiber shows three components clearly, that are, a center porous medulla, a middle layer of cortex and a thin surface layer of tiles-overlapping scales (around 0.5 *μm* of thickness). Three animal hair fibers under dry state confirm the structure as shown the SEM images in Fig. 3a.

Their difference displays at the configuration that is resulted from the volume ratio and texture of cortex and medulla. Medulla is a kind of biodegradable cellular materials in the form of porous medium that benefits the hair warmth retention. Cortex accounts for the major body of hair and undertakes the main stretch in deformation. In fact, the two-phase model for hair fiber simplifies the structure of cortex using crystalline phase and matrix. The swelling takes place evidently at cortex after hair fiber soaked in aqueous environment, as shown in Fig. 3a. In comparison, the cortex swells thicker after penetration of aqueous molecules into the matrix phase along with the narrow of center medulla than the dry cortex. On the basis of the two-phase model^30^,^31^, the matrix phase is in amorphous state with a large amount of loose polar groups that can interact with aqueous molecules. This enlarges the distance of neighboring polypeptide chains by disrupting HBs between the groups of N-H and C=O at adjacent branches. From the macroscopic point of view, the volume of cortex is increased for diametral swelling with more circular cross section.

The diametral swelling corresponds to the quasi-plastic distortion by means of altering the matrix phase at the weak attractive groups on the macromolecule branches. Straightforward from Fig. 3b, the innate camel hair displays straight smooth configuration along the hair axis. A manual deformation of the hair with entanglement onto a circular bar and immersion of the deformed hair into water result in the hair fiber with plasticized and wrapped on the bar. A drying process gives rise to the hair in temporary spatial profile. To large extent, the spatial deformation recovers to the straight shape after immersion of it in water for a few minutes. The detailed shape recovery dynamics of a deformed hair encountered with water can refer to the supplementary material Scheme-1. In comparison, the recovered camel hair and innate hair reveal almost the same shape without any spatial residues.

### Water-induced SME quantification of hair fibers

Figure 4a shows that the three animal hairs have the measured *R*_*f*_ values more than 0.9, indicating the good hydration (penetration of aqueous molecules) and fixation (removal of aqueous molecules) related to the HBs at the original and dislocated macromolecules, respectively. The *R*_*f*_ value is increased as the increase of set folded angles, indicating the less effect of folding process on the macromolecules network when the set angle is enlarged. The highest *R*_*f*_ value of camel hair, compared with other two hairs, may have the most normalized amounts of HBs in the camel hair cortex for the best temporary shape fixation. Figure 4b shows the goat hair with the highest *R*_*r*_ value for the best SME ability. The relationship of *R*_*r*_ and *θ*_*s*_ also displays the increasing tendency for both parameters for three hair fibers. *R*_*r*_ correlates with the entropic stress resulted from the net-points and connected network. The lowest *R*_*r*_ value of sheep hair may imply the weakest net-points for the recovery force, indicating the number and type of net-points in the current test hair may be the least. The thinnest cortex as shown in Fig. 3a is evidenced for the cause of lowest *R*_*r*_ value of sheep hair. In contrast, rapid recovery of goat and camel hair fibers are normally five seconds faster compared to the sheep hair, show the strong recovery stress resulted from the net-points on the basis of the large amount or density of either crystalline phase or DBs.

Cyclic tensile to hair fibers can also reflect the SME ability of different hair fibers by means of strain recovery under water dropwise to the stretched hair. The set tensile strain (0.10) is beyond the yield point of hair (~0.05) that the shape memory part can be identified from the elastic and permanent unrecovered strains. In Fig. 4c, the elastic strain is the instant recoverable strain released when the stretched hair begins to backward the original point whereas the unrecovered strain can be found at the onset of the second tensile curve. In comparison of tensile curves of Fig. 4c, sheep hair also accounts for the largest unrecovered strain after water stimulation while goat hair still has slightly less unrecovered strain than camel hair, being consistent with the test results as shown in Fig. 4b. At strain of 0.10, goat and camel hairs show remarkably larger tensile loads than sheep hair, supporting the viewpoint of stronger networks and net-points of these two hair fibers. Consistent with the single *R*_*r*_ values at *θ*_*s*_ = 0 for three hairs, Fig. 4d displays the related *R*_*r*_ values at *θ*_*s*_ = 0 for seven cycles of SME programs, which indicates that the shape recovery ability of animal hair stimulated by water is almost invariable (decreasing less than 5%). This indicates the stable SME performance of animal hair using water as a stimulus.

Under wet state, hair fiber usually shows significantly lower stress than dry hair at the same tensile strain for the cause of disrupted HBs. The relaxation takes place for the clamped condition of hair fibers with rapid decay of loading at the initial stage, followed with a decrease rate of loading and reached a stable state. The relaxation is ascribed to the structure variation of networks between net-points. Oppositely, upon unloading, the external load is zero during steady-state relaxation of present shape recovery process. As shown in Fig. 5a, an initial strain of *ε*_0_ is given to the dry and wet hair fibers for stress-free recovery. The dry and wet hair fibers are from the two parts of a hair fiber where one part is for dry stretching and dry recovery and the other is for wet stretching and wet recovery. A dramatic decrease of recovery time is noted for stretched hair fiber by encountering with water than at dry condition. By taking the viscoelasticity into account, the shape recovery kinetics can be quantitatively interpreted and compared in terms of relaxation time. On the basis of the two-phase model, a single relaxation unit is visualized as a parallel connection of an elastic spring and a viscous dashpot according to the Kelvin-Voigt model^41^, as illustrated in the inset of Fig. 5b. Thus, a relationship is given for the strain and recovery time,


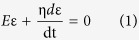


here, ε and t denote the strain and recovery time, E is the elastic modulus of spring and η is the viscosity of dashpot, respectively. The remaining strain at any recovery time can be obtained following the form of Kohlrausch-Williams-Watts relaxation formula as,


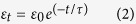


where τ = η/E is termed as the relaxation time and *ε*_0_ is the initially applied strain^42^.

Both the involved strain and relaxation time reflect the weighted averages of those all relaxation units. Accordingly, a linear formula can be derived as,


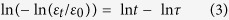


Which can be verified by the good linearity of experimental results, as shown in Fig. 5b. The value of τ was fitted to be 2.42 × 10^3^ s and 18.2 s for dry and wet samples, respectively. This indicates that the hydration (on the amorphous HBs) remarkably reduces the relaxation time and narrows the relaxation spectrum for the shape recovery process of the present hair fiber^43^. Moreover, the linearity of recovered strain and time from Fig. 5a,b indicates the uniform recovery velocity of stretched hair in water^43^. The hysteresis of dashpot neutralizes the sine nature of pure spring recovery velocity that manifests the linear fitting of strain versus recovery time, further ahead, the larger slope of the linearity implies the higher recovery velocity that is consistent within the both figures.

Dynamic mechanical analysis (DMA) was performed on the camel hair, as shown the result in Fig. 5c, that the storage modulus increased significantly when temperature reached a high level which implies that new bonds were generated in the dried state of the hair. The short period of temperature rise up to 85 °C for drying the camel hair in Fig. 5c was extended remarkably under room temperature that the storage modulus undergoes rapid rise, following with slowed down of rise rate, as shown the modulus rise curve in Fig. 5d. In Fig. 5c, the wetting process of the dry camel hair endows the hair with rapid decrease of storage modulus in linearity, which is in contrast to the tendency of rise stage along with the increasing temperature. The decreased modulus indicates the significant aqueous plasticization on the camel hair, implying that the penetrated aqueous molecules destroyed certain bonds in the hair amorphous region^44^. Evident repeated cycling of storage modulus with the rise and down of temperature suggests the bonds open and close in reversible nature. In Fig. 5d, the monotonically increase of storage modulus by 30% and the markedly decreased loss factor to the original 60%, tan *δ*, in the drying process of wet camel hair, indicate a notable enhancement of recoverable elasticity by dehydration accompanied by lowering in hysteresis energy dissipation (i.e. loss modulus is decreasing). This suggests that the dynamic mechanical properties of animal hair fibers are strongly sensitive to the aqueous stimuli, and such trend can be corroborated by the variations of moduli in several synthetic SMPs with different constituents^45^,^46^,^47^.

### Identification of net-point and switch unit of hair fibers

The structural differences between dry and wet hair samples have been evaluated from a few experimental aspects. As indicated by the arrows in Fig. 6b, the diffraction shoulders and peaks arise on the XRD pattern by hydration with relative sharp peaks become slightly weakened for goat and camel hairs, especially at the abscissa of 2θ = 9°(0.98 *nm*) and 21°(0.46 *nm*). The relative weak detection of the characteristic peak at 2θ = 9° for sheep guard hair may indicates scarcely crystalline phase existed inside the tested hair body, thus the hydration has hardly effect on the hair characteristic peaks. The change of XRD pattern from dry to wet conditions indicates a slight decrease in the content of crystalline component or certain destruction of microscopic ordered arrangement in hair^48^. Conversely, the existing of XRD peaks of hair in wet state indicates its net-point role in hair SME performance. Moreover, the drying process to the wet hairs rearranges the destructed microscopic crystalline component into ordered consequence, as shown the reappeared sharp peaks in the dried hair from the attached supplementary material (Scheme-2).

With respect to the comparison of hair fiber for Raman spectra under dry and wet conditions, it is noted that the ‘dry’ and ‘wet’ curves can be viewed as almost coincident for each animal hair in the Raman scanned regions (abscissa values of Fig. 6c). Specifically, symmetrical DB mode from 500–580 cm^−1^ can be found as a broad characteristic peak which is associated with several molecule conformations^49^,^50^, i.e. *g-g-g (510* *cm*^−1^), *g-g-t (525* *cm*^−1^) and *t-g-t (540* *cm*^−1^) (*g* and *t* denote *gauche* and *trans*) conformations. The Raman spectra of hairs from dry to wet, then to dry conditions can refer to Scheme-3. Fig. 6c suggests that aqueous molecules have ignored effect on the DBs in hair, indicating this chemical crosslinking may act as net-point in hair SME behaviors. In Fig. 6d, a broad absorption band at around 3400 cm^−1^ corresponding to free water is introduced to the ATR-FTIR curve of wet sample^51^. In particular, both the characteristic peaks of C=O stretching (Amide band I) and N-H bending (Amide band II) vibrations are shifted to higher wavenumbers from 1624 cm^−1^ to 1628 cm^−1^ and 1517 cm^−1^ to 1532 cm^−1^, respectively, as shown the inset in Fig. 6d. This implies that intermolecular HBs are formed between the residues and aqueous molecules during the hydration, which is consistent with the results reported previously^45^,^46^,^52^. Therefore, the absorbed aqueous molecules within biopolymer hair exist in two distinct states of free water and bind water.

**Figure 1:**
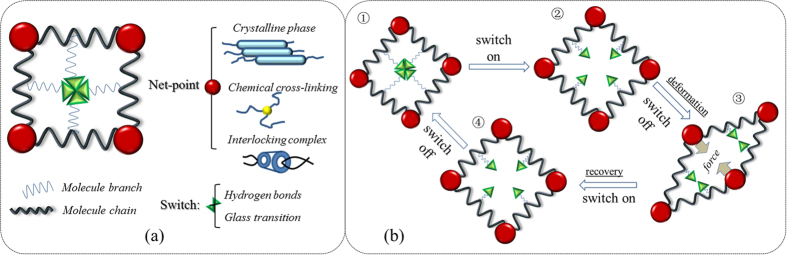
(**a**) a structural model of SMP consisting of net-point and switch unit in which the former may be composed of crystalline phase, chemical crosslinkers, or interlocking supermolecular complex while the latter may be hydrogen bonds or glass transition, (**b**) a cycle of shape memory program for the structural model responsive to a stimulus, in detail: ➀ the innate shape of the structure model, ➁ the switch is opened by a stimuli environment, ➂ an external force to deform the structure model and switch off for the temporary shape fixation, ➃ memorized shape among net-points and switch off to return the original shape when encountering with the stimuli environment.

**Figure 3:**
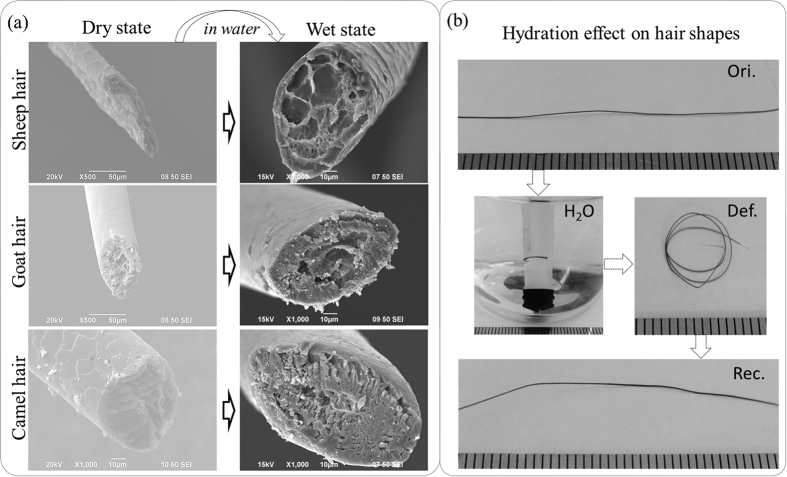
Hydration effect on animal hairs: (**a**) SEM images of cross sections of sheep, goat, and camel hair fibers under dry and wet states; (**b**) demonstration of water sensitive SME using appearances of a camel guard hair at its original state, after temporary shape fixation using hydration process, and shape recovery encountering with water; Note: all figures were obtained under room temperature.

**Figure 4:**
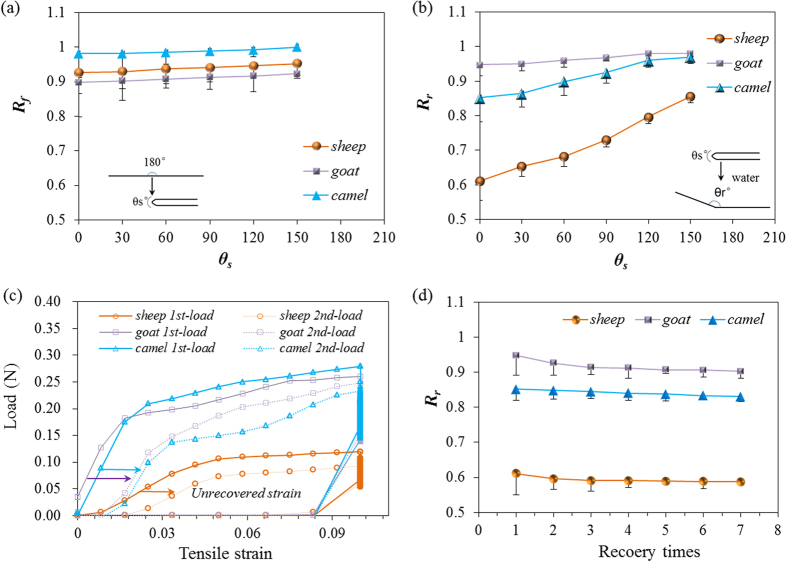
Quantification of water-stimulated SME of three hair fibers: (**a**) fixation ratio (*R*_*f*_) in temporary shape corresponding to a set of folded angles (*θ*_*s*_); (**b**) shape recovery ratio (*R*_*r*_) from temporary shape to the innate shape, the inset illustrates the process from *θ*_*s*_ to *θ*_*r*_ of a camel hair encountered with water. (**c**) two tensile cycles of three hair fibers induced by water in the shape recovery stage; (**d**) shape recovery ratios of three animal hairs from *θ*_*s*_ = 0 to the innate shape with the cycle number of SME program.

**Figure 5:**
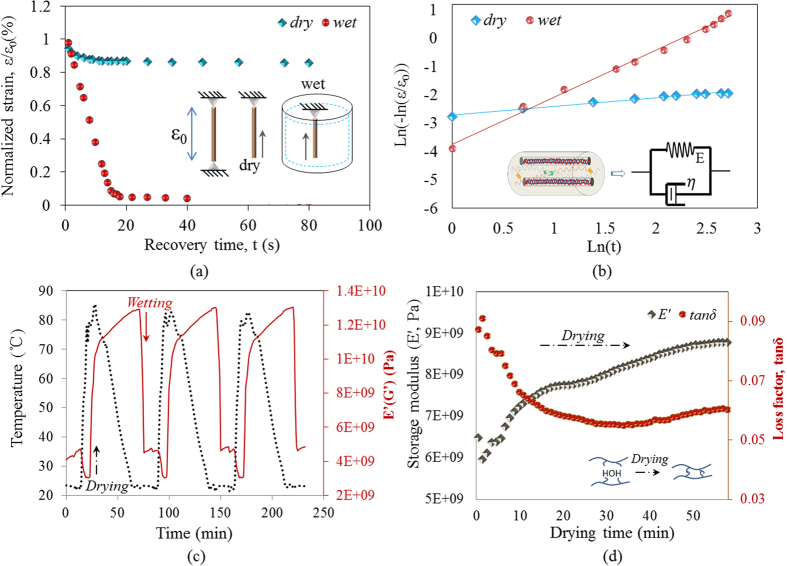
Shape recovery kinetics: (**a**) variations of remaining strain *ε*_*t*_ normalized by the initial applied one *ε*_o_ as a function of recovery time *t* under dry and wet conditions for camel hair fiber. The illustration of the stretched hair fiber in recovery under dry and wet conditions is presented in the inset; (**b**) shape recovery process of dry and wet samples analyzed using the kinetic model; (**c**) cycles of temperature rise and down between 20 °C and 85 °C (dehydration) for investigating variation of storage modulus of camel hair during water immersion followed using DMA; (**d**) variation of storage modulus (*E*’) and loss factor (tan *δ*) of camel hair as a function of drying time under room temperature using DMA, the inset shows the cause of increase of *E*’ during dehydration with the increase of HBs.

**Figure 6:**
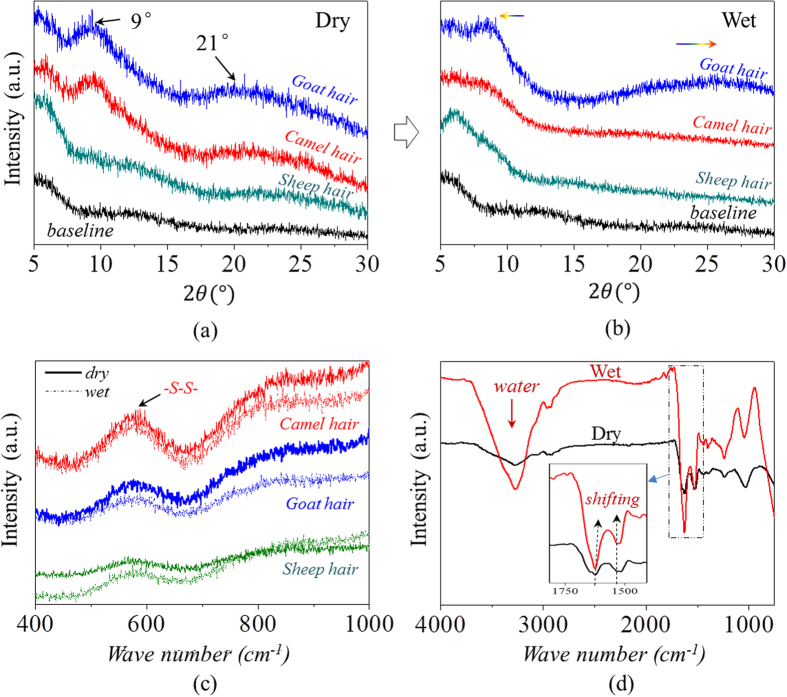
Structural component characterization: XRD raw data of three animal hair fibers under (**a**) dry and (**b**) wet conditions on the basis of baseline, (**c**) Raman spectra for characterizing the DBs of three animal hair fibers under dry and wet conditions, (**d**) FTIR characteristic peaks of goat hairs under dry and wet status where the shifting of polar groups is presented in the inset of the spectra.

**Figure 7:**
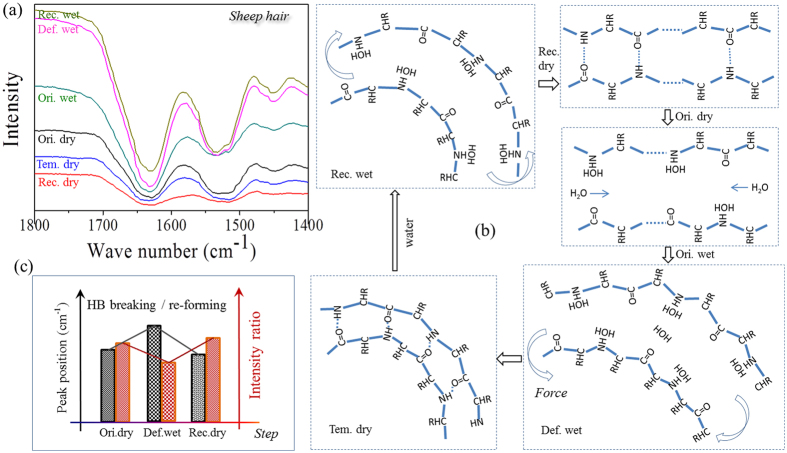
The work mechanism of switch unit in water-sensitive SME of hairs: (**a**) FTIR results of a sheep hair in SME characteristic steps; (**b**) schematic illustration of effect of aqueous molecules on HBs between hair keratin macromolecule chains in amorphous area; (**c**) summary of IR characteristic peaks representing HBs in switch on and off using wave number shifting and ratio variation of peak intensity; (**d**) disruption and re-formation of HBs from IR peak shifting.

In detail, from the viewpoint of shape memory program according to Fig. 1b, six ATR-FTIR curves for the case of sheep guard hair are given in Fig. 7a that correlate to each SME step as labeled in the figure. Besides, it is noted that the intensity ratio of characteristic peaks of N-H bending to C=O stretching vibrations are evidently different for the hair in dry and wet conditions. The wavenumber shifting and varied intensity ratio of two characteristic peaks between three key SME steps are listed in Table 1 for the three typical animal hairs. In respect of wavenumber shifting, characteristic peak of C=O stretching is increased by 4 cm^−1^ to 7 cm^−1^ from dry to wet states, in turn, the wavenumber is decreased by 4 cm^−1^ to 8 cm^−1^ from wet to dry states. In similarity, characteristic peak of N-H bonding is increased and decreased by shifting 5 cm^−1^ to 15 cm^−1^ and 5 cm^−1^ to 16 cm^−1^, respectively. This reversible shifting related to the conversion between dry and wet conditions suggests that the intermolecular HBs undergo the reversible destruction and formation processes, accordingly^39^.

Furthermore, Table 1 gives the change of intensity ratio between the two characteristic peaks of three animal hairs, identically, in which it is found that the decrease and increase of the ratio from the hair states of dry (Ori. dry) to wet (Def. wet) and wet (Def. wet) to dry (Rec. dry) reverses respectively. This can be interpreted from the schematic illustration of Fig. 7b on the basis of molecule motion viewpoint, which also corresponds to the six steps of water-sensitive SME program, that we consider pairs ‘Ori. dry’ and ‘Rec. dry’, ‘Def. wet’ and ‘Rec. wet’ should be identical from the ATR-FTIR scan curves. Table 1 gives that the intensity ratio of ‘Rec. dry’ step almost equals one for three hair samples, which means the intensities of characteristic peaks at 1620~1640 cm^−1^ and 1510~1535 cm^−1^ are observed to equal each other, indicating the same amount of carbonyl group (C=O^…^) and imino group (N-H^…^) approximately. The decreased peak intensity ratio related to the wet hair state indicates that the penetrated aqueous molecules disrupt the hydrogen bond formed by carbonyl and imino groups. Each aqueous molecule was attached to each imino group for the polar attraction between the atoms hydrogen (N–H) and oxygen (H-O-H), as shown the schematic steps ‘Ori. wet’ and ‘Def. wet’ in Fig. 7b. This leads to the decreased number of discrete amino groups for 1510~1535 cm^−1^ and the reduced intensity ratio between the two characteristic groups. Removal of and encountering with water to the hair enable the same interaction to take place among the atoms of hydrogens and oxygens on the groups of amino, carbonyl and hydroxyl in hair matrix, as shown the schematic steps from ‘Tem. dry’ to ‘Rec. wet’ and ‘Rec. wet’ to ‘Rec. dry’ in Fig. 7b. It should be pointed out that the interaction of polar molecules between the macromolecule chains only demonstrates the transformation process of each SME step, the interaction dynamics (speed of switch on and off in macroscopic) is not involved in the illustration.

In summary of Fig. 7(a,b), two characteristic peaks of animal hairs appearing at 1620~1640 cm^−1^ and 1510~1535 cm^−1^ of ATR-FTIR spectra undergo the higher wavenumber shifting and decreased peak intensity ratio from the process of hair samples in dry and original state to deformed and wet state, and vice versa, as shown the summary of tendency in Fig. 7c. The wavenumber shifting and regular variation of peak intensity indicate that HBs in animal hairs can lock the hair temporary shape by removal of its internal water and recover to its innate shape by encountering with aqueous environment. This reversible cycle of hydrogen bond implies its switch unit role in water-sensitive shape memory effect.

### Water-sensitive SME mechanism for animal hairs

**Figure 8:**
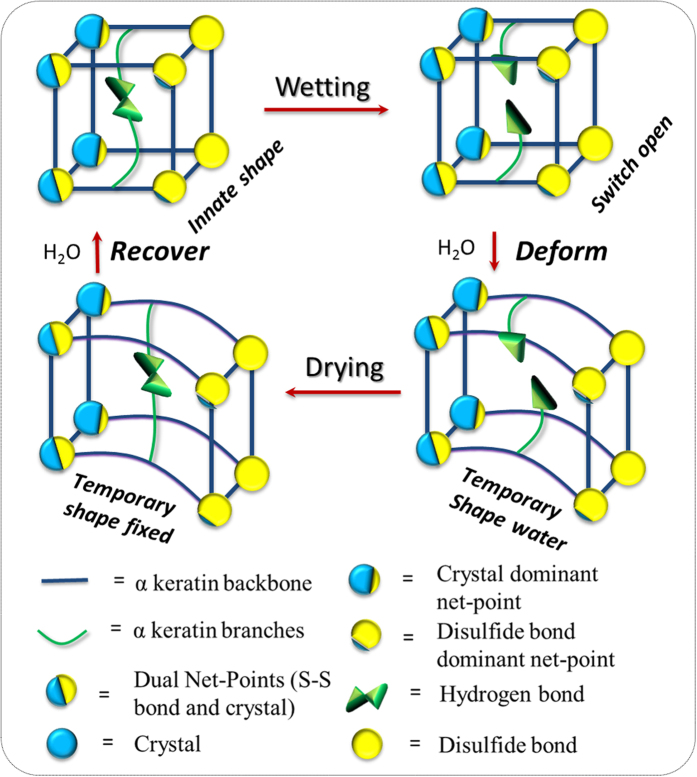
A structural model for water-sensitive shape memory mechanism of animal hairs.

Through the above analyses of variation of hair structure in water-induced SME program, a SME structural model is proposed to illustrate the related mechanism for biopolymer hair fiber, as shown in Fig. 8. The model suggests that animal hairs have the same switch unit in water-sensitive SME, i.e. HB (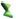). The wetting process of hair causes the HBs destructed in the hair matrix area, resulting in the hair with a lateral swelling and enhanced flexibility of polypeptide chains for the cause of enlarged distance of keratin backbones and branches by aqueous polar molecules. In water, the hair can be given any temporary shape and then the pairs of HB residues are dislocated from the original positions. A drying process to the temporary shape of hair fibers removes the free aqueous molecules inside the hair body, leaving the HB residues on the neighboring macromolecule branches to form new HBs for fixing the deformed hair. The stress between net-points linked by networks is stored by the new HBs. However, it would release if the new HBs encounter with aqueous molecules again. From the macroscopic viewpoint, the deformed hair recovers to its innate shape in water.

In the model, the switch unit is framed by net-points that are connected by keratin macromolecule backbones. There are two kinds of net-points for the animal hairs, i.e. crystals and DBs. During the hydration process of hair, aqueous molecules have insufficient effect on the net-points so that the net-points can maintain the hair structure intact. From different types, ages, body parts of animals, hairs may have different contents of crystalline phase and DBs. For instance, in the above structural component analyses, the employed sheep hair has little crystalline phase, thus, its SME can be regarded as DB-dominant net-points (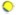) for water-sensitive SME. The other two hairs both demonstrate high *R*_*r*_ value for the reason of crystalline phase and DB act as net-points, namely, twin net-points (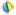). Some animal hairs may have little content of cysteine, thus, their water- sensitive SME may be ascribed to the crystal- dominant net-points (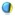). Moreover, it is found that the stronger and more number of net-points in the hair would give rise to the better water-sensitive SME, which may inspire to design more remarkable SME of man-made polymers^53^,^54^. The structural model proposed here may suggest mechanism for processing optimization on animal hair fibers in relation to wet conditions and offer new functions in textiles or the applications such as human hair treatment. In addition, if a reduction and/or oxidation process is involved on animal hairs, the SME structural model of the animal hairs will be different in relation to the open and close of DBs, thus new models need to be established which is in progress and will be presented in our coming publications.

### Research summary

In this work, three typical animal hair fibers from sheep, goat and camel were investigated on their water-responsive behavior and corresponding mechanisms in molecular and morphological structural networks. The innate shape of a natural biopolymer animal hair can be recovered through hydration after deformation fixed, which is a typical water-stimulated SME of a smart material. High temporary shape fixation (>90%), original shape recovery (>60%) and repeatable memory cycles by drying and wetting processes demonstrated that animal hairs are smart α-keratin fibers. The shape recovery kinetics can be quantitatively described in terms of linearity of recovery strain against time according to the viscoelastic relaxation model. Single and cyclic tensile tests on dry and wet hairs reveal that the hydration can reduce the hair modulus and stretching force significantly. The remarkably quick recovery stimulated by water is ascribed to the notably lowered modulus by hydration due to the improved motion flexibility of macromolecular chains as a result of swelling effect and broken hydrogen bonds by water. In contrast, the drying process to the wet hair can reversibly increase the hair elastic modulus resulted from locking the macromolecular chains in terms of hydrogen bonding. The exploration into structural components (crystal, disulfide covalent and hydrogen bonds) reveals a SME structural network mechanism model in macromolecular assemblies of such smart fibers. Thereafter, a hybrid structure network consisting of single-switch (HBs) and twin-netpoint (crystals and DBs) is identified in their structures. It is inferred that more remarkable SME of a hair is due to stronger net-points in the biopolymer networks in which crystals and DBs can work together or separately while HBs act as the switch unit. This single-switch and twin-netpoint structural model can vary in different hair types for α-keratin biopolymers and realize different levels of shape memory ability. This study is expected to provide inspiration for making more remarkable synthetic shape memory polymers.

## Materials and Methods

### Preparation of hair fibers

Three raw hair fibers from adult sheep, goat and camel’s back were purchased from a trade factory (Sunite Right HTC villi LLC, Mongolia Autonomous Region, China). The hair samples were firstly combed to remove the mud and impurities between fibers, then, they were screened to the diameter more than 50 μm (guard hairs) for the following study. The samples were soaked in the ethanol/chloroform mixed solution for removing the surface fatty materials^55^. The removal procedure of the fatty film from the animal hair surface using the organic solvents was continued only for one minute, which was assumed to have negligible effect on the hair cortex and medulla. The removal of the fatty film conduced to the efficient interaction of hair cortex with aqueous molecules in the later performance test. Then the samples had two rinses by distilled water, and they were dried at constant 40 °C atmosphere in an oven for characterizations. The moisture regain of hair fibers were measured in the range of 12% and 15%.

### Qualitative study of SME

The conditioned straight hair fibers were immersed in water at 20 °C for twelve hours to ensure the full interaction of matrix phase (phase M) of hair fibers with aqueous molecules. Under water, the hair fibers were wrapped on a circular bar manually, and maintained the shape for two hours to endow the fibers with full plasticization. The hair fibers were then taken out of water and dried at room temperature for one day. The hair shape in each process, including the temporary shape for investigating the shape fixation ability, was observed using an optical camera. When the entangled spiral dry fibers in temporary shape encountered with water, the shape recovery behaviors were recorded. This method can refer to the work^25^.

### Quantitative characterization of SME

The shape memory ability of hair fibers was studied quantitatively according to Fig. 1b with five usual steps. Instead of temporary entangled spring shape fixation, variation of triangle from folded hair fiber into a certain angle *θ*_*s*_ based on the transition of wet and dry states is used for investigating the hair SME ability. The ideal SME shows a process of ‘*θ* = *180°* *→* *θ* = *θ*_*s*_*°* *→* *θ* = *180°*’ under penetration and removal of aqueous molecules into and out of hair fibers. However, for most natural fibers, the ideal full shape fixation and recovery cannot be realized for the cause of non-consistent network between net-points along the fiber axis. There are two variables thus come across for justifying the hair SME ability, that are, *θ* = *θ*_*f*_*°* and *θ* = *θ*_*r*_*°*, representing the shape fixation angle and recovered angle respectively. Shape fixation ratio (*R*_*f*_, Eq. 4) and shape recovery ratio (*R*_*r*_, Eq. 5)^6^ are thereafter derived on the basis of the two measured angles,


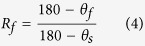



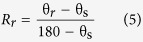


Physically, a greater *R*_*f*_ value means a higher sensitive switch to be on and off, whereas a higher value of *R*_*r*_ implies a better SME ability of hair fiber. During SME investigation of each fresh sample, the angles of *θ*_*f*_ and *θ*_*r*_ are required five times of measurement for the average values. The related *R*_*f*_ and *R*_*r*_ values are calculated with standard derivations. Cyclic tensile of animal hairs is consistent with the variation of angle measurement for studying the hair SME ability. The test employs an Instron machine (5566) with the load cell of 5 *N* in the measure scope. The unrecovered strain (ε_1_) from the first stretch will be partially recovered (to ε_2_) when the stretched hair sample is encountered with water. In this case, the shape recovery ratio^6^,^56^ of hair fiber is calculated according to Eq. 6,


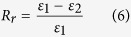


### Identification of net-point and switch unit

The surface scales, cross-section morphologies and diametral swelling of three animal hair fibers were gold-coated and observed using an environmental scanning electron microscope (SEM, JEOL Model JSM-6490) before and after soak in water for six hours. Over the SME investigation, the chemical functional groups and intermolecular bonds of hair fibers were examined using Fourier Transform Infrared Spectroscopy (PerkinElmer Spectrum 100 FT-IR Spectrometer, USA) in the scan range of wave numbers between 3500~650 *cm*^*−1*^ using ATR (Attenuated-Total-Reflectance) method. The absorption spectra were recorded with eight scans at a resolution of 16 *cm*^*−1*^. The angle (ϕ) of incidence light was adjusted to 39°, ATR crystal was diamond (refractive index *n*_1_ is 2.4), and the refractive index of hair fiber (*n*_2_) is around 1.5. The characterized depth of penetration (*d*_*p*_) is in the range of 1~15 *μm* according to Eq. 7:


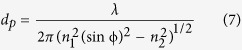


here, λ is the wavelength of light. Raman spectra yield similar but complementary information to the Infrared spectroscopy. It relies on Raman scattering from a laser in the near infrared range. The light interacts with molecular vibrations, resulting in the energy of the laser photons being shifted up and down. The chemical cross-links such as disulfide bond (DB) of hair fibers under the SME investigation were characterized by a Horiba Jobin Yvon HR800 Raman spectrometer, which was equipped with an *Ar* laser (λ = 448 *nm*, 180 *mW*) as the excitation light source, and an Olympus BX41 microscope. The water-impenetrable phase C according to the two-phase model can be characterized by X-ray diffraction due to the Bragg regular arrangement of crystalline phase. Thus, the crystallinity of hair fibers at dry and wet conditions were determined by Rigaku Smart Lab XRD system (9 KW) that is equipped with Cu Kα radiation with a wavelength of *1.54* 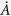. The hair fibers were minced in the format of short chips (powder) to cover the stage. The test 2θ range is from *5°* to *30°* and recorded at a scan speed of *10°∙min*^*−1*^ at *40* *kV* and *40* *mA*. The structural analysis of hair fibers in dry and wet conditions using the above characterizations were conducted for individual hair samples that were from the two parts of one hair fiber.

## Additional Information

**How to cite this article**: Xiao, X. and Hu, J. Animal Hairs as Water-stimulated Shape Memory Materials: Mechanism and Structural Networks in Molecular Assemblies. *Sci. Rep.* **6**, 26393; doi: 10.1038/srep26393 (2016).

## Supplementary Material

Supplementary Information

## Figures and Tables

**Figure 2:**
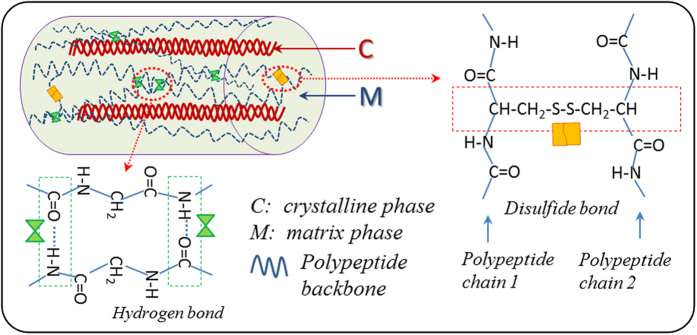
Schematic illustration of cylindrical two-phase model of a wool fiber consisting of water-impenetrable rods (phase C) set parallel to the fiber shaft embedded in the matrix (phase M) which is available to be weakened by water, and hydrogen and disulfide bonds between two neighbor polypeptide chains.

**Table 1:** Characteristic IR peaks at key steps of water-sensitive SME.

Goat hair	*Ori. Dry*	1624	1517	95.86%
	*Def. Wet*	1628	1532	87.18%
	*Rec. Dry*	1624	1516	98.28%
Camel hair	*Ori. Dry*	1627	1524	97.26%
	*Def. Wet*	1631	1532	93.11%
	*Rec. Dry*	1627	1520	99.65%
Sheep hair	*Ori. Dry*	1626	1520	98.35%
	*Def. Wet*	1633	1525	85.34%
	*Rec. Dry*	1625	1520	99.44%
State states		IR peak position/Wave number(cm^−1^)		Peak intensity ratio
