# Supplementary material for: Animal Hairs as Water-stimulated Shape Memory Materials: Mechanism and Structural Networks in Molecular Assemblies
Source: Sci Rep. 2016 May 27;6:26393. doi: 10.1038/srep26393 (PMC4882536; doi:10.1038/srep26393)
Supplement: Supplementary Information [file srep26393-s1.pdf]

Animal Hairs as Water-stimulated Shape Memory Materials: Mechanism and Structural Networks in Molecular Assemblies

Xueliang Xiao [1,2], Jinlian Hu [2*]

[1] *School of Textiles and Clothing, Jiangnan University, Wuxi, 214122, P.R. China*
[2] *Institute of Textiles and Clothing, the Hong Kong Polytechnic University, Hong Kong*

*Corresponding author:
Tel.: +852-27666347; Fax: +852-27731432; E-mail: tchujl@polyu.edu.hk (J.L. Hu)

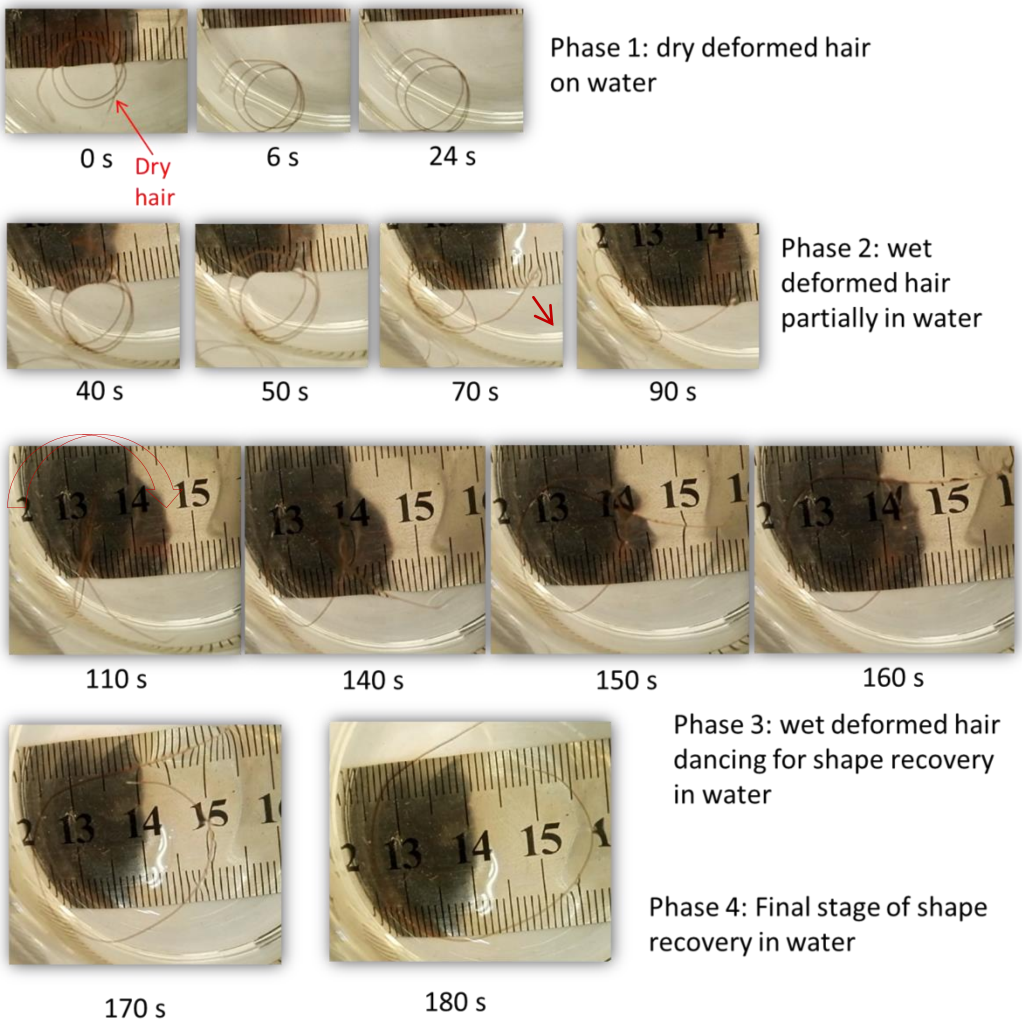

Scheme-1 Shape recovery dynamics of deformed hair in water

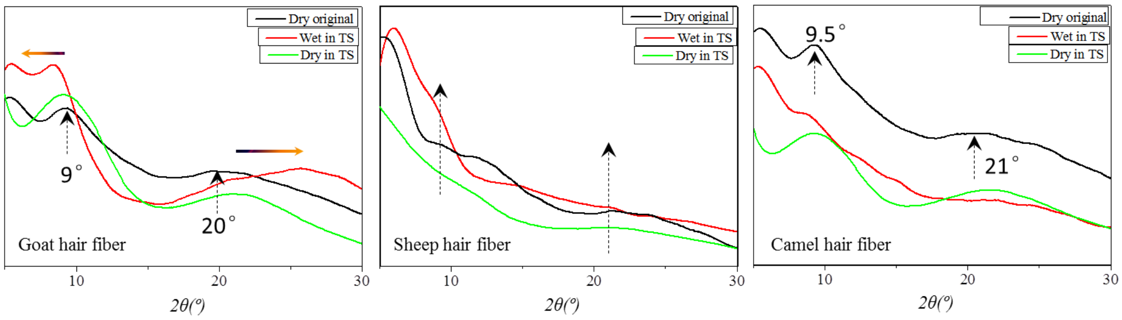

Scheme-2 XRD raw data (manually smoothed) of three hair fibers at three shape-memory characterized statuses ('-original' means dry straight hair, '-wet TS' means hair in water with temporary shape, '-dry TS' means dry hair in temporary shape)

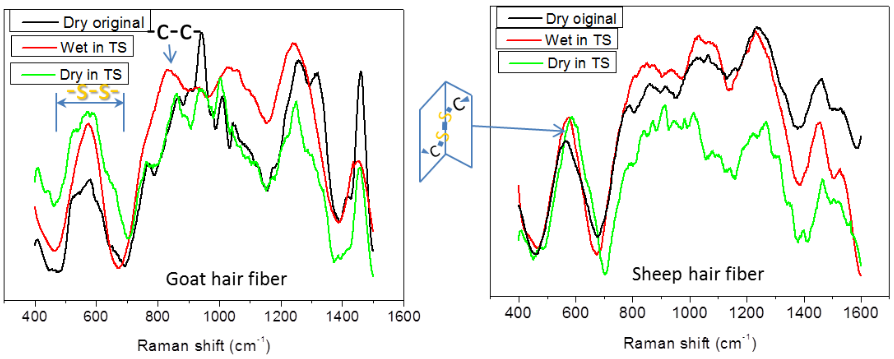

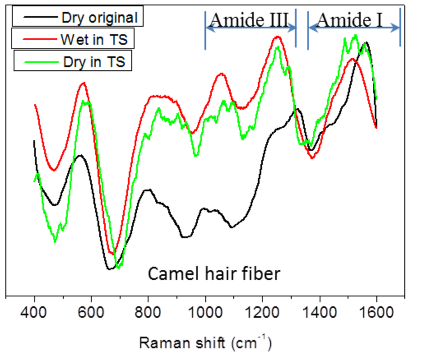

Table Raman characteristic bands and their assignments

Amide band	Band position (cm^{-1})	Band assignment
Amide I	1500~1600	β-pleated sheet, α-helix, $CO\text{-}NH_2$
Amide III	1000~1350	α-helix, Disordered, β-pleated sheet
-C-C- Skeletal	800~950	α-helix
-S-S-/ -C-S-	500~600/ 600~650	-S-S- -C-S-

Scheme-3 Raman shifts (manually smoothed) of three hair fibers at three shape-memory characterized statuses ('-original' means dry straight hair, '-wet TS' means hair in water with temporary shape, '-dry TS' means dry hair in temporary shape), and characteristic band positions and related assignments
